# *Lactiplantibacillus plantarum* 0111 Protects Against Influenza Virus by Modulating Intestinal Microbial-Mediated Immune Responses

**DOI:** 10.3389/fmicb.2022.820484

**Published:** 2022-06-30

**Authors:** Jun-Hong Xing, Chun-Wei Shi, Ming-Jie Sun, Wei Gu, Rong-Rong Zhang, Hong-Liang Chen, Ying Li, Dan Wang, JunYi Li, Tian-Ming Niu, Qun-Tao Huang, Jia-Hao Qian, Hai Bin Huang, Yan-Long Jiang, Jian-Zhong Wang, Xin Cao, Nan Wang, Yan Zeng, Gui-Lian Yang, Wen Tao Yang, Chun-Feng Wang

**Affiliations:** ^1^Key Laboratory of Animal Production and Product Quality Safety of Ministry of Education, College of Veterinary Medicine, College of Animal Science and Technology, Jilin Provincial Engineering Research Center of Animal Probiotics, Jilin Agricultural University, Changchun, China; ^2^Shandong BaoLai-LeeLai Bioengineering Co., Ltd., Tai’an, China

**Keywords:** probiotics, influenza virus, IFN-β, T cells, gut microbiota

## Abstract

There are some limitations of traditional influenza vaccines concerning novel mutant strains. Therefore, it is particularly important to develop preventive means for antigen-unrelated types of influenza viruses. Recent studies have shown that probiotics can modulate the immune system and reduce the severity of viral infections. In this study, we investigated the potential of *Lactiplantibacillus plantarum* 0111 against influenza virus H9N2. Challenge experiments showed that *L. plantarum* 0111 pretreatments could effectively improve mice’s survival rate and weight loss and reduce the inflammatory cytokines IL-6 and TNF-α in the lungs and bronchoalveolar lavage fluid (BALF) along with the degree of lung and intestinal injury. FMT experiment demonstrates that the protective effect produced by *L. plantarum* 0111 is associated with gut microorganisms. In addition, 16S high-throughput sequencing of the mouse intestinal microbiota showed that *L. plantarum* 0111 remodeled the intestinal microbiota after H9N2 infection and maintained the gut microbiota balance. In a mouse model, the oral administration of *L. plantarum* 0111 increased IFN-β expression in the serum and BALF. At the same time, the transcript levels of IFN-β and related ISGs in the intestine and lungs of mice were also increased. In addition, the activation and polarization of T cells in mesenteric lymph nodes (MLNs) and the spleen were detected by flow cytometry, and the results showed that *L. plantarum* 0111 modulated cytokines in T cells and increased IgA expression in B cells in the MLNs and spleen. Thus, *L. plantarum* 0111 may improve gut microbiota-mediated immune responses and thus, resist infection by the influenza virus, and it could be used as an effective preventive measure against the influenza virus.

## Introduction

According to disease severity, avian influenza viruses are divided into low and highly pathogenic viruses (LPAIs and HPAIs). Some LPAIs, such as the H9N2 subtype, can transmit between birds, mammals, and humans, which usually causes economic losses to the breeding industry and poses a serious threat to human health (Peiris et al., 1999; Zhang et al., 2013, 2020). In 1998, human infection with the H9N2 subtype influenza virus was first discovered in southern China (Guo et al., 1999), followed by increasing case reports of H9N2 infection of humans (Song and Qin, 2020) and even cases of death due to infection with this virus (Peacock et al., 2019). Viruses directly infect humans while providing heavy ligands for some or even all of the internal genes of highly pathogenic influenza viruses, such as H7N9 (Pu et al., 2015), which pose a significant threat to public health. H9N2 can replicate in respiratory and gastrointestinal cells, and through pathological damage, immunosuppression causes damage to the host (Yao et al., 2014; Zhang et al., 2014; Li et al., 2016; Zhu et al., 2018). Vaccination against AIV is usually a practical preventive approach, but it is not enough to completely resist viral invasion because viral mutations occur quickly (Ujike et al., 2010). Therefore, it is essential to study and develop new therapeutic approaches to cope with emerging heterologous influenza viruses.

The World Health Organization defines probiotics as live microorganisms that confer a health benefit to the host when administered in adequate amounts. In addition, probiotics can effectively promote the health and growth of host animals by regulating the balance of microorganisms in the intestinal tract, enhancing the barrier function of intestinal epithelial cells, inhibiting the growth and adhesion of pathogenic microorganisms, and regulating immunomodulatory effects (Bubnov et al., 2015; Hu et al., 2017; Cao et al., 2020). Lactic acid bacteria are the most common probiotics and have been widely used and considered safe for humans and animals (Slattery et al., 2019). The metabolites they produce, such as organic acids and bacteriocins, have beneficial effects on the host (Yousefi et al., 2019). In addition, lactic acid bacteria stimulate and regulate the immune system of the intestinal mucosa, protecting in the event of pathogenic and viral infections of the organism (Surendran Nair et al., 2017). A previous study showed that oral administration of *Lactobacillus pentosus* increased IgA production, thereby protecting mice from lethal influenza virus infection (Kobayashi et al., 2011). At the same time, probiotics also have side effects. Probiotic consumption is associated with D-lactic academia and acidosis in adults and infants on probiotic-containing formula, and d-lactic acidosis is the culprit for brain fogginess (Papagaroufalis et al., 2014; Rao et al., 2018).

The gut microbiome functions include nutritional effects, metabolic effects, and immune stimulation (Sánchez et al., 2017). It has been shown that H9N2 avian influenza virus infection causes disturbance of the host gut microbiota (Li et al., 2018). After the gut microbiome imbalance, it cannot effectively play the physiological role of a normal gut microbiome, damages the body’s immune function, and reduces the body’s clearance of influenza virus (Pang et al., 2018). At the same time, oral probiotics help establish a mutually beneficial symbiotic intestinal micro-ecological balance system (Hu et al., 2017). Therefore, it is important to explore probiotics to regulate intestinal flora dysregulation caused by the influenza virus.

*Lactiplantibacillus plantarum* 0111, a natural strain, was screened from the intestine of mice and had high immune activity and adherence. In the present study, the oral administration of *L. plantarum* 0111 improved survival and reduced body weight loss in infected mice. In addition, *L. plantarum* 0111 modulates the intestinal microbiota and the immune system and could be a candidate strain to prevent influenza virus infection.

## Materials and Methods

### Bacteria, Cells, Viruses, and Animals

*Lactiplantibacillus plantarum* 0111 was kindly provided by the Shandong Baolai Lilai Bioengineering Co., Ltd., NCBI accession number: MT907445, using MRS medium. After 16 h of anaerobic culture at 37°C, the bacteria were centrifuged at 4000g for 10 min at 4°C, washed three times with sterile phosphate-buffered saline (PBS, pH = 7.4), and then resuspended in PBS for subsequent application. Madin-Darby canine kidney (MDCK cell line) cells were preserved in this experiment and cultured with DMEM, FBS, 10,000 U/ml penicillin, and 10,000 μg/ml streptomycin at 37°C in 5% CO_2_. To ensure cell viability, the cells were recovered before use and passaged for more than three generations.

H9N2 was preserved by our laboratory (Shi et al., 2014). The median tissue culture infective dose (TCID_50_) was used as the titer level for the influenza virus. SPF grade 6-week-old C57BL/6 female mice (*n* = 90, five in each cage) were purchased from the Beijing Vital River Laboratory Animal Technology Co., Ltd. These mice were allowed free access to water and food in SPF Animal Farming Center. All animal experiments met the requirements of the 113 Animal Management and Ethics Committee of Jilin Agricultural University (JLAU20200704001).

### Experimental Design and Sample Collection

*Lactiplantibacillus plantarum* 0111 (10^8^) was gavaged in a volume of 200 μl for 7 consecutive days, and the control group was gavaged with PBS. Experiment groups were challenged with H9N2 (10^6.5^ EID_50_) on day 0. The mice were anesthetized by the intraperitoneal injection of sodium pentobarbital, and H9N2 was infected by nasal drops (Yang et al., 2018). All mice were monitored every day for 14 days, and their body weight and survival rate were recorded. Fecal samples are collected on day 7 and immediately stored at −80°C for total DNA extraction and sequencing. In another repeat experiment, after 7 days of gavage of *L. plantarum* 0111, serum and bronchoalveolar lavage fluid (BALF) were collected from mice to detect type I interferon, and the lungs and jejunum of mice were collected for qPCR analysis. A single-cell suspension was prepared for flow cytometry analysis in the spleen, Peyer’s patches, and MLNs (Jiang et al., 2017; Yang et al., 2018). the lungs and jejunum of mice were collected on day 7 post-infection, fixed in 4% formaldehyde for histopathological analysis. Lung tissue and BALF were prepared to detect viral titers and inflammatory cytokines. BALF was inserted into the trachea using a 1 ml sterile syringe and fixed, while 800 μl of cold PBS was injected, repeatedly withdrawn 15 times, transferred into a 1.5 EP tube, and centrifuged at 2,000 rpm for 10 min; the supernatant was frozen at −80°C.

### Antibiotic Treatment and FMT

To eliminate as much as possible the intestinal microbial background of mice, ampicillin (1 mg/ml), streptomycin (5 mg/ml), vancomycin (0.25 mg/ml), and mycomycin (1 mg/ml; Solarbio) were added to the sterile drinking water of mice for 6 days, followed by FMT. Mouse feces from the PBS + H9N2 and 0111 + H9N2 groups were collected on the seventh day and processed as described previously (Yang et al., 2021). Briefly, fecal samples were placed in a sterile N_2_ incubator, mixed thoroughly, and then plugged into a cell filter to collect bacteria and centrifuged at 12,000 rpm for 10 min. The samples were subsequently added to 10% glycerol, stored at −80°C. FMT before replacing the glycerol with PBS and stained with methylene blue staining to count the number of viable microorganisms. Mice were gavaged in 100 μl volumes (*n* = 5). One day later, all mice were intranasally attacked with H9N2 (10^6.5^ EID_50_), and their survival and weight loss were monitored 14 days after infection. FMT experiment was repeated twice independently.

### Illumina Sequencing

A 16S rRNA sequencing library of the V3–V4 hypervariable region was constructed according to a previous study in our laboratory (Chen et al., 2020). See S Methods for specific methods. The primer sequences were as follows: forward primer 5′-GTACTCCTACGGGAGGCAGCA-3′; reverse primer 5′-GTGGACTACHVGGGTWTCTAAT-3′. This sequence has been logged into NCBI (SRP301018). Raw data sequencing first removed barcode and primer sequences, then spliced them to obtain raw tags. Raw tags were used to remove chimeras and short sequences to obtain clean tags. Sequences were subjected to a classification operation (cluster). The sequences were classified into many OTUs according to their similarity through the classification operation. Sequences were typically OTU partitioned at a 97% similarity level, followed by bioinformatics statistical analysis of the OTUs. Finally, α diversity, β diversity, and different species screened were analyzed according to OTU and a taxonomic rank.

### Flow Cytometry Analysis

To determine the cellular immune response induced by *L. plantarum* 0111, we euthanized mice on day 0 and subsequently collected spleens, Peyer’s patches, and MLNs and prepared single-cell suspensions. FACS analysis was performed using PerCP-Cy5.5-labeled anti-mouse CD3 antibody, PE-Cy7-labeled anti-CD4 antibody, APC-Cy7-labeled anti-CD8 PE-labeled anti-IFN-γ antibody, APC-labeled anti-IL-4 antibody, FITC-labeled anti-TNF antibody, APC-labeled anti-B220 antibody, and FITC-labeled anti-IgA antibody. All antibodies were purchased from Becton, Dickinson, and Company in the United States. Experiments were performed three times, and data compensation and analysis were performed using FlowJo v7.6.1. In parallel, IFN-α and IFN-β expression in serum and BALF at day 0 and IL-6 and TNF-α expression in the lung supernatant and BALF on day 7 were analyzed using the MU Anti-Virus Response Panel (13-plex, LEGENDplexTM, 740,622) according to the manufacturer’s instructions, and data analysis was performed using LEGENDplex v8.0.

### Virus Titer Detection

To determine the virus titer, an equal weight of lung tissue was homogenized in DMEM to obtain a serially diluted 10-fold suspension of tissue homogenate. Moreover, it was titrated in 96-well culture plates of MDCK cells. The Reed-Muench method was adopted to calculate the titer and was expressed as log_10_TCID_50_/g of lung tissue.

### Quantitative PCR

According to the manufacturer’s instructions, we extracted total RNA from the mouse intestine and lung using a Universal RNA Extraction Kit (Takara). Reverse transcription was performed using TransScript^®^ One-Step gDNA Removal and cDNA Synthesis SuperMix (TRANSGEN China). Triple qPCRs were subsequently performed in an Applied Biosystems 7,500 (Life Technologies, United States of America) using PerfectStart^™^ Green qPCR SuperMix (TRANSGEN China). Thermal cycling conditions were 94°C for 30 s, followed by 40 cycles of 95°C for 5 s and then 60°C for 30 s. Data were collected using an Applied Biosystems 7,500 (Life Technologies, United States of America) and analyzed by the threshold cycle (ΔΔCT) method using 7,500 Software v2.3. The qPCR primers are designed to avoid contamination of the genome by cross-intron design, thereby inhibiting the amplification of genomic DNA. Primers are shown in Supplementary Table S1.2.

### Histological Analysis

Mouse lung tissues and jejunum were fixed in 4% formaldehyde and subsequently embedded in paraffin. Tissues were dehydrated and stained in graded alcohols and then sectioned. At least two tissue sections (3 μm) from each sample were stained with hematoxylin–eosin (HE) and finally analyzed using an inverted fluorescence microscope (Leica Microsystems, Germany) in our laboratory.

### Statistical Analysis

All data were expressed as the mean ± SEM. Statistical analysis was performed using one-way ANOVA and *t*-tests with GraphPad Prism 8.0 (GraphPad Software). The *p* values are indicated as follows: ^*^p < 0.05; ^**^p < 0.01; and ^***^p < 0.001.

## Results

### The Protective Effect of *Lactiplantibacillus plantarum* 0111

**Figure 1:**
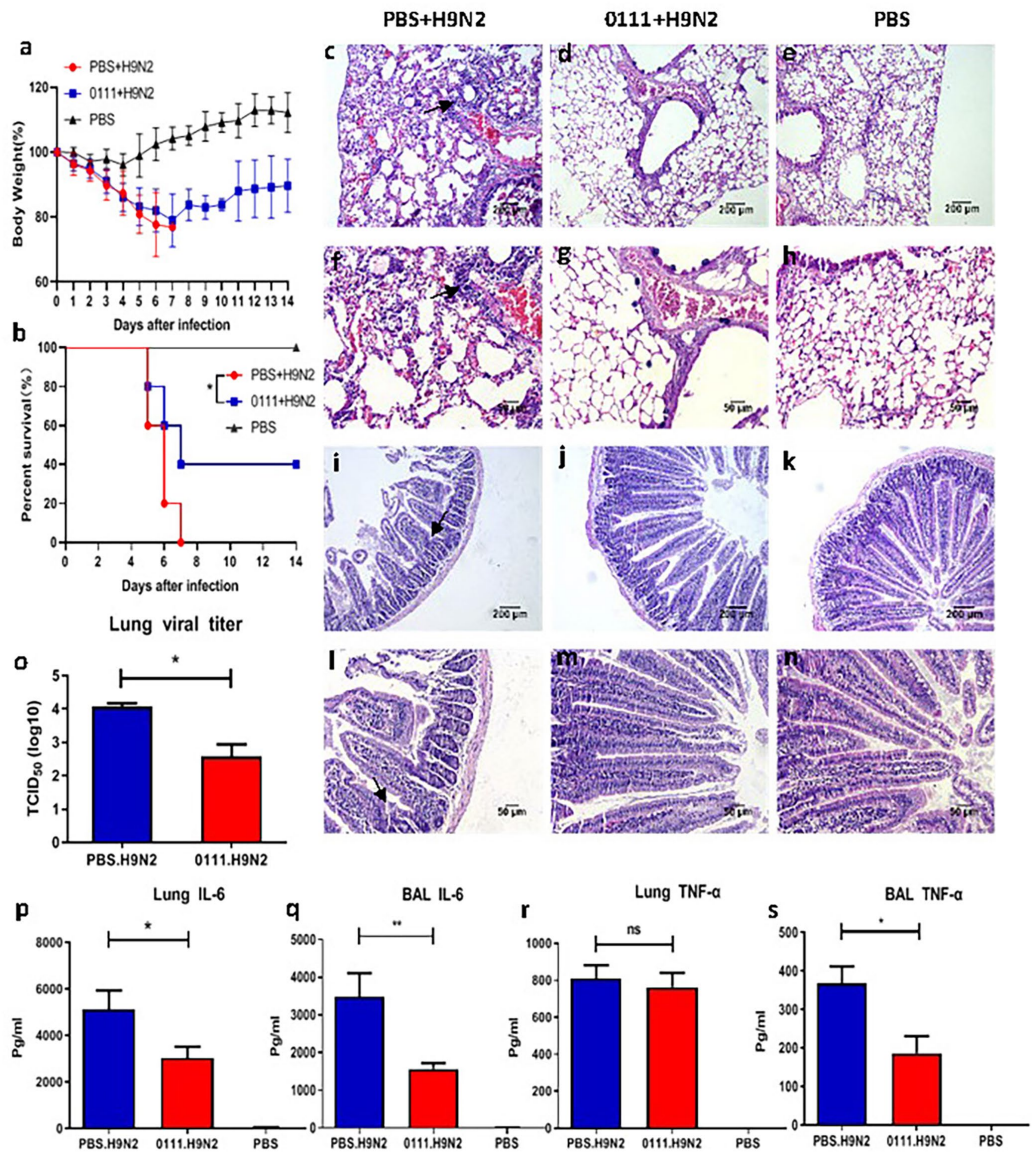
Pretreatment of C57BL/6 mice with *Lactiplantibacillus plantarum* 0111 reduced weight loss, improved survival and reduced lung and jejunum tissue damage as well as reduced lung viral load and inflammatory cytokines caused by H9N2 influenza virus infection. C57BL/6 mice (*n* = 5/group) were infected 7 days after the continuous oral administration of H9N2 virus (2 x LD50) by *L. plantarum* 0111 (1 × 108 CFU/200 glimouse). The body weight **(A)** and survival rate **(B)** of the mice are recorded within 14 days after infection. On day 5 after infection, some mice were euthanized, and the lungs and jejunum were collected and histologically analyzed. Lung viruses and inflammatory cytokines were measured in the lungs and bronchoalveolar lavage fluid (BALF). **(C,D,E,I,J,K)** magnification at 100×. The scale bar indicates 200 μm. **(F,G,H,L,M,N)** magnification at 200×. The scale bar indicates 50 μm. **(C,F)** the lungs of mice were challenged with H9N2 after PBS pretreatment; **(D,G)** the lungs of mice were challenged with H9N2 after *L. plantarum* 0111 pretreatment; **(E,H)** the lungs of mice were challenged with PBS only. **(I,L)** mouse jejunum challenged with H9N2 after PBS pretreatment; **(J,M)** mouse jejunum challenged with H9N2 after *L. plantarum* 0111 pretreatment; **(K,N)** mouse jejunum treated with PBS only. **(O)** pulmonary virus log_10_TCID_50/g_ lung tissue. **(P)** IL-6 (pg/ml) in lungs. **(R)** TNF-α (pg/ml) in lungs. **(Q)** IL-6 (pg/ml) in BALF. **(S)** TNF-α (pg/ml) in BALF. The results are presented as the means ± SEMs (*N* = 5), and statistical significance was calculated by one-way ANOVA. ^*^P < 0.05; ^**^P < 0.01.

We determined 108 CFU as the optimal dose (Supplementary Figures S1B,E). Subsequently, mice were challenged with the H9N2 subtype avian influenza virus and examined for health status over 14 days. After the viral challenge, mice usually died between day 5 and day 8 after infection (Figure 1B). The 0111 + H9N2 group provided 40% more protection against H9N2 attack than the PBS + H9N2 group (p < 0.05). In terms of body weight, except for the control group, the bodyweight of mice in the other groups gradually decreased after being challenged, and by day 7, the 0111 + H9N2 group showed a trend of gradual weight regain and reached more than 80% by day 14 (Figure 1A). In contrast, the PBS + H9N2 group continued to decline until day 7. Therefore, L. plantarum 0111 pretreatment improved survival and weight loss in mice.

Histological analysis of the lungs and jejunum of mice from each group showed that the H9N2 influenza virus caused damage to the lungs and the jejunum. Foamy exudates and damaged alveolar structures were observed in mice from the PBS + H9N2 group (Figures 1C,F). The alveolar structures were intact, and lung damage was not present in the 0111 + H9N2 group (Figures 1D,G). Meanwhile, the jejunal wall of mice in the PBS + H9N2 group (Figures 1I,L) was thickened, intestinal villi were shed, and inflammatory cells in the lamina propria were infiltrated compared with the PBS group (Figures 1K,N). However, no damage state was found in the 0111 + H9N2 group (Figures 1J,M). Thus, *L. plantarum* 0111 pretreatment could mitigate the lung and jejunum injury caused by H9N2.

Five days after infection with H9N2, the mice were euthanized to prepare lung homogenates, and the supernatant was inoculated into MDCK cells to determine the TCID_50_ (Figure 1O). After viral infection, mice in the 0111 + H9N2 group had approximately 1.6-fold lower virus than mock-treated mice (*p* < 0.05). In the meantime, inflammatory cytokines are produced excessively during influenza infection, and inflammatory cytokine storms can cause severe lung damage. Therefore, the measurement of IL-6 and TNF-α inflammatory cytokines in the lungs and BALF could help understand the mechanism of lung injury caused by H9N2. At 5 days post-infection, the levels of IL-6 detected in the lungs (*p* < 0.05) and BALF (*p* < 0.01) were significantly lower than those in the PBS + H9N2 group (Figures 1P,Q). In addition, the level of TNF-α in the BALF (*p* < 0.05) was also significantly lower than that in the PBS + H9N2 group, while no difference was produced in the lungs (Figures 1R,S). *L. plantarum* 0111 decreased the production of proinflammatory cytokines induced by H9N2 infection.

### The Protection Provided by *Lactiplantibacillus plantarum* 0111 Is Associated With the Gut Microbiota

**Figure 2:**
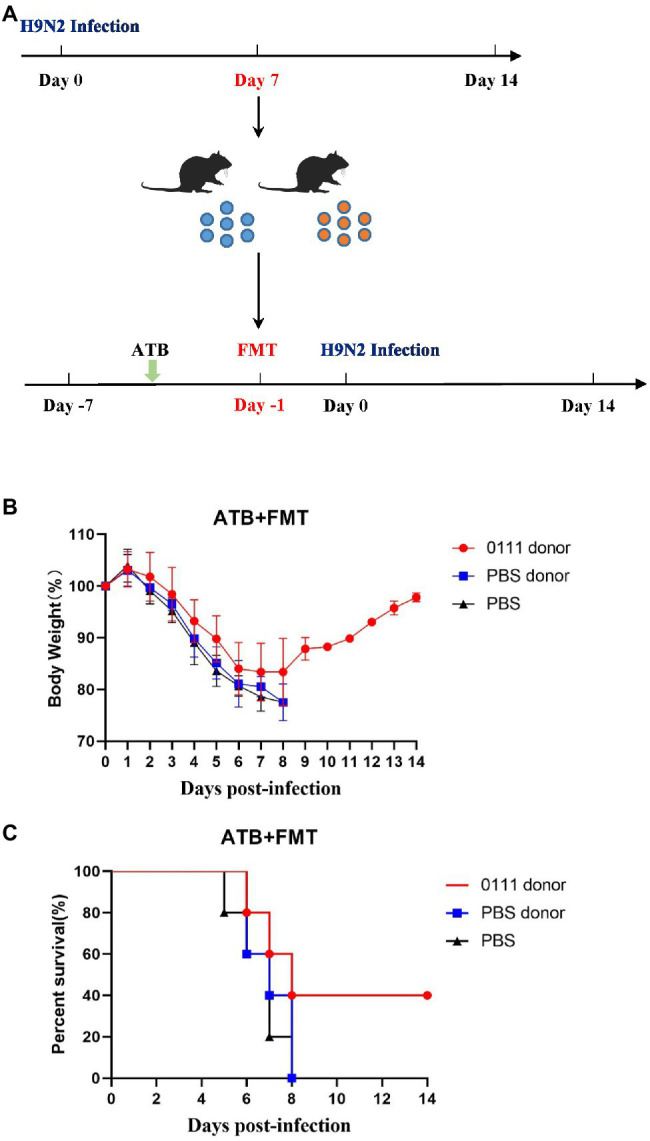
FMT experiment. Mice feces were collected on day 7 after challenging H9N2 and FMT was performed 6 days after antibiotic treatment **(A)**. Mice (*N* = 10/group) were challenged on day 0 and were observed for the next 14 days for infection. Body Weight in mice receiving FMT **(B)**. Survival rate of mice receiving FMT **(C)**.

*Lactiplantibacillus plantarum* primarily colonizes the intestinal tract and exerts a probiotic effect. To verify whether the protective effect of *L. plantarum* 0111 is associated with gut microorganisms, we performed FMT on mice (Figure 2A). The result showed that mice treated with the 0111 + H9N2 group improved survival and weight loss, while mice treated with the PBS + H9N2 group showed the same susceptibility as the control group (Figures 2B,C), demonstrating the protective effect of *L. plantarum* 0111 through the gut microbiota.

### α and β Diversity of Gut Microbial Composition

Based on the sequencing analysis of the 16S rRNA gene, a total of 2,894 OTUs were obtained from 15 samples with a Good’s coverage index greater than 98%, and the Good’s coverage of all samples (Supplementary Figure S3A), rarefaction curves (Supplementary Figure S3B), Shannon–Wiener index (Supplementary Figure S3C) and species accumulation (Supplementary Figure S3D) curves indicated sufficient data sampling and sequencing depth. The database of 16S rRNA gene sequences almost completely covered all microbial communities.

**Figure 3:**
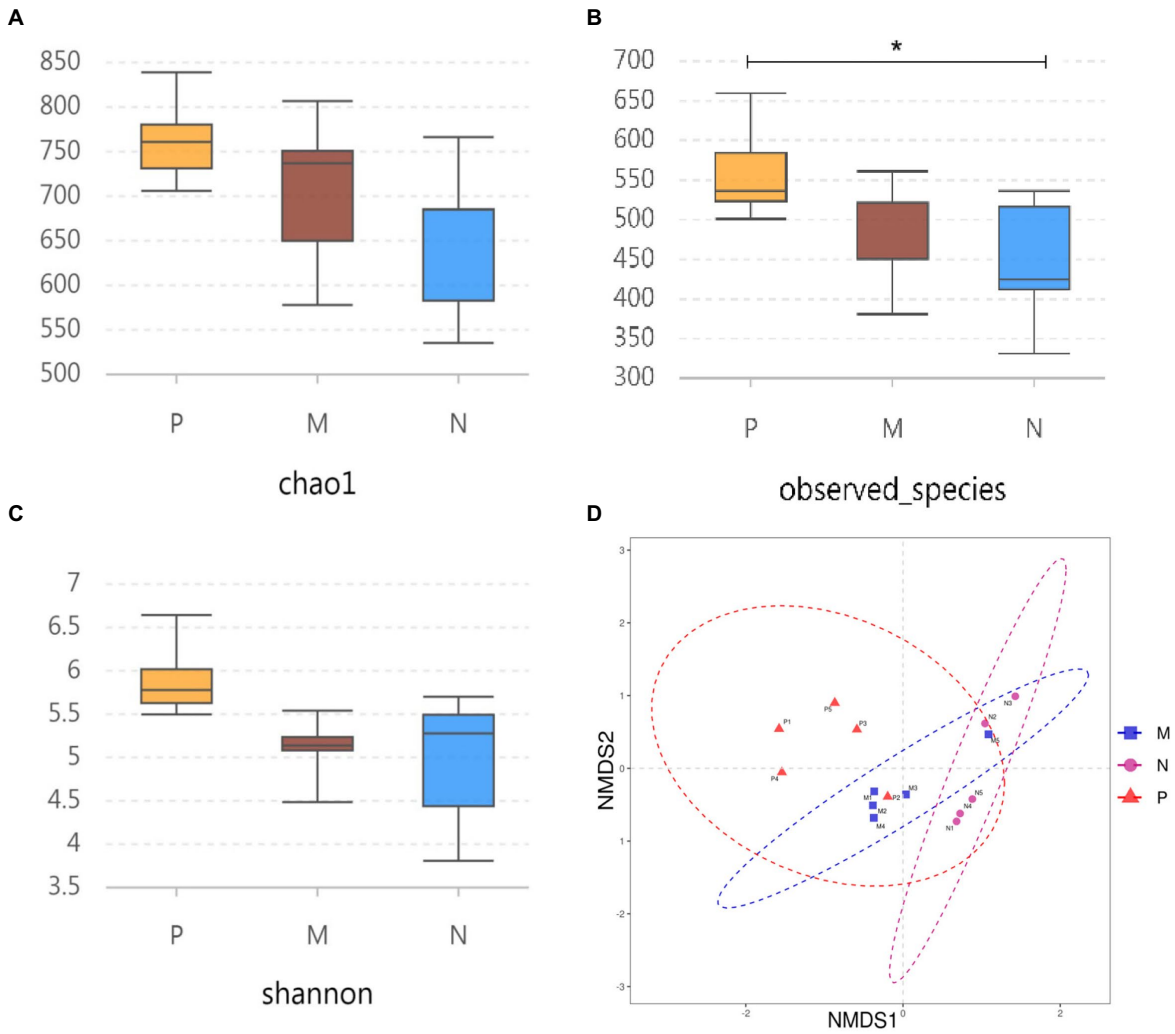
α diversity analysis and β diversity of the three experimental groups were analyzed by sequencing the 16S rRNA gene. Chaol **(A)** and the observed number of species **(B)** were used as abundance estimates. The Shannon—Wiener index **(C)** was used as a diversity estimator. **(D)** NMDS was adopted to analyze the degree of difference between different samples to assess β diversity. The results were calculated for statistical significance using the *t*-test (*N* = 5/group). ^*^P < 0.05. P, challenged H9N2 cells after pretreatment with PBS; M, challenged H9N2 cells after pretreatment with *L. plantarum* 0111. N, pretreatment with PBS.

The α diversity was evaluated using the Chao1, observed species, and Shannon indices. The results showed that H9N2 infection significantly increased the number of actually observed OTUs compared with the PBS group (*p* < 0.05), while the number of OTUs was not disturbed in the oral 0111 group (Figure 3B). In addition, although the Chao1 and Shannon indices were not significantly different, there was an upward trend compared with the PBS group, whereas the 0111 group tended to be stable (Figures 3A,C). In the meantime, NMDS (nonmetric multidimensional scaling) was used to analyze the β diversity in samples from different groups. In striking agreement with the α diversity, H9N2 infection caused significant differences in biological populations compared with the PBS group, whereas no significant differences were observed in the 0111 group (Figure 3D). Therefore, oral administration of *L. plantarum* 0111 can effectively maintain the intestinal imbalance caused by H9N2 infection.

### *Lactiplantibacillus plantarum* 0111 Remodels the Gut Microbiota After H9N2 Infection

**Figure 4:**
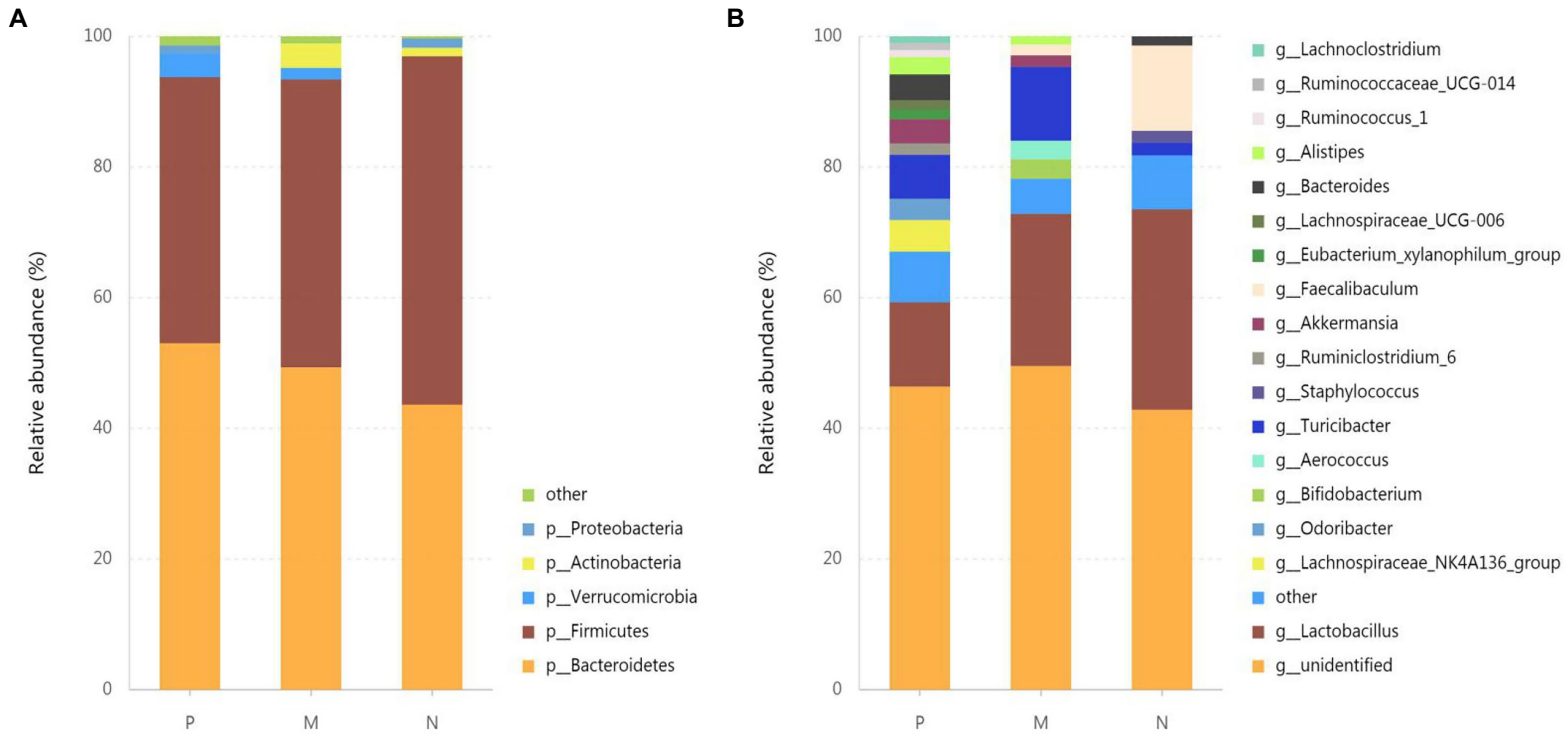
Effect of *L. plantarum* 0111 on the gut microbial composition in mice infected with H9N2. Relative abundances of gut microbial groups at the phylum **(A)** and genus **(B)** levels. The relative abundances of gut bacteria presented here were calculated by averaging the data obtained from five replicates in each group. P, challenged H9N2 cells after pretreatment with PBS; M, challenged H9N2 cells after pretreatment with *L. plantarum* 0111; N, pretreatment with PBS.

To investigate the effect of *L. plantarum* 0111 on gut microbial composition in mice infected with H9N2, we analyzed bacteria at the phylum (Figure 4A) and genus (Figure 4B) levels to characterize the dynamics of microbial taxonomic distribution. Proteobacteria, Actinobacteria, Verrucomicrobia, Firmicytes, and Bacteroidetes predominate in gut microbial communities at the phylum level. *Proteobacteria*, *Actinobacteria*, *Firmicytes*, and *Bacteroidetes* accounted for 1.44, 1.29, 53.31, and 43.62% in group N, respectively. At the same time, *Actinobacteria* did not appear in group P, and *Actinobacteria* and *Bacteroidetes* increased to 3.672 and 53.02%, respectively. The proportion of *Firmicytes* decreased to 40.717%. *Actinobacteria*, *Verrucomicrobia*, and *Bacteroidetes* did not increase in group M, accounting for 3.73, 1.75, and 49.32%, respectively, while *Firmicytes* decreased to 44.08%.

*Lactobacillus* and *Faecalibaculum* were important commensal microbiota that maintains intestinal homeostasis, which accounted for 30.66 and 13.05% of *Lactobacillus* and *Faecalibaculum*, respectively, at the genus level in group N. However, *Faecalibaculum* was not observed, and *Lactobacillus* accounted for only 12.92% in group P. At the same time, *Lactobacillus* and *Faecalibaculum* accounted for 23.28 and 1.64% of group M, respectively. Interestingly, *Lachnoclostridium*, *Ruminococcaceae-UCG-014*, *Ruminococcus-1*, *Lachnospiraceae-UCG-006*, *Eubacterium-xylanophilum*-group, *Ruminiclostridium-6*, *Odoribacter*, and *Lachnospiraceae-NK4A136*-group appeared only in group P.

### LEfSe Analysis of Landmark Commensal Species

**Figure 5:**
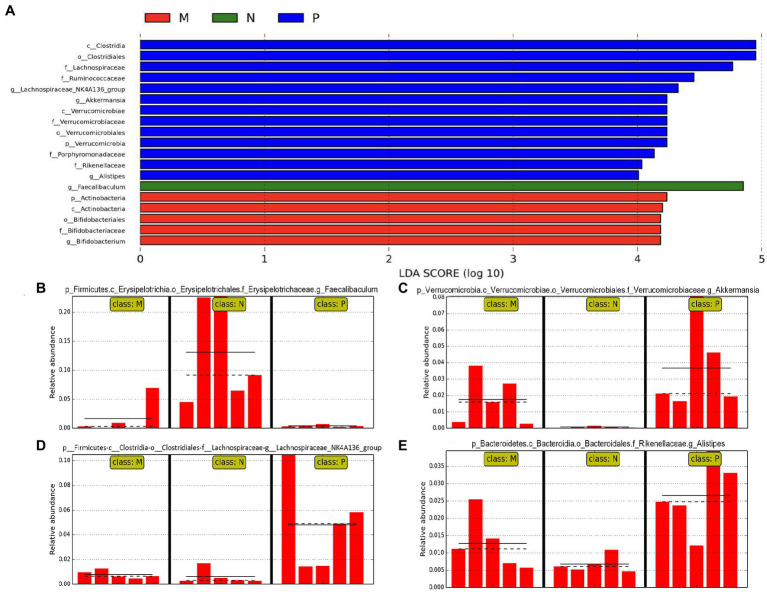
*Lactiplantibacillus plantarum* 0111 increased the abundance of *Faecalibaculum* to maintain gut balance. LEfSe analysis was performed to assess changes in gut microbes. **(A)** LDA scores obtained in LEfSe analysis of gut microbiota from different groups. A LDA effect size of greater than 3 was used as a threshold for the LEfSe analysis. **(B)** *Faecalibaculum*, **(C)** *Akkermansia*, **(D)** *Lachnospiraceae*-NK4A136-group, and **(E)** *Alistipes* abundance analysis in different groups. P, challenged H9N2 cells after pretreatment with PBS; M, challenged H9N2 cells after pretreatment with *L. plantarum* 0111; N, pretreatment with PBS.

To fully understand the effect of *L. plantarum* 0111 on the gut microbiota of H9N2-infected mice, we performed LEfSe (LDA effect size) analysis. The LEfSe branch diagram (Supplementary Figure S4) and LDA scores (Figure 5A) were used to assess changes in gut microbes. LEfSe analysis showed that *Faecalibaculum* (Figure 5B) was significantly decreased in group P compared with group N, while it tended to rise in group M. In comparison, Akkermansia (Figure 5C) and Alistipes (Figure 5D) were significantly increased in group P. Still, it correspondingly decreased in group M. At the same time, *Lachnospiraceae-NK4A136*-group was significantly increased in group P compared with group N (Figure 5E), and showed normal levels in group M. Therefore, *L. plantarum* 0111 can maintain intestinal homeostasis after H9N2 infection by interacting with commensal microorganisms.

### *Lactiplantibacillus plantarum* 0111 Increases the Expression of IFN-β and ISGs

**Figure 6:**
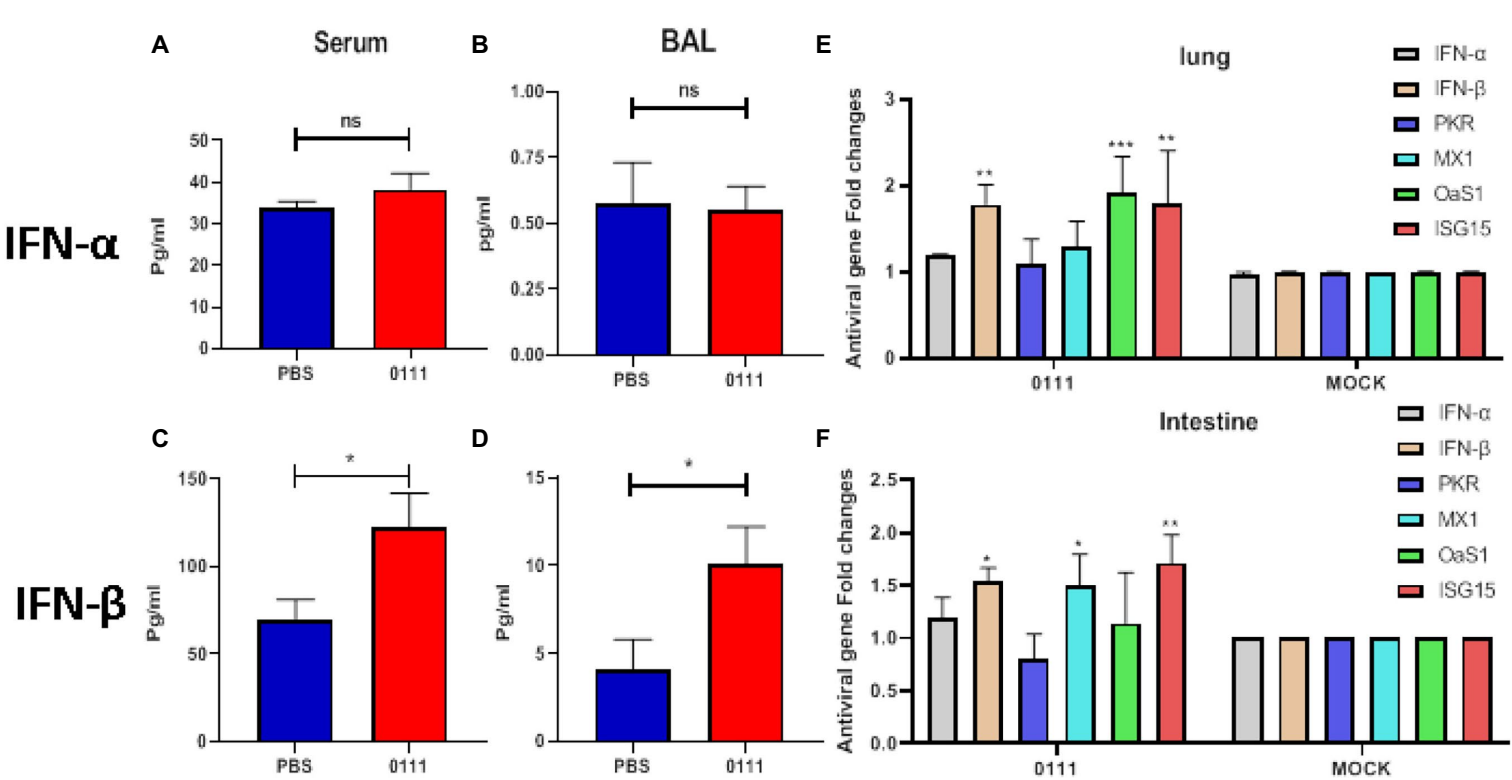
Pretreatment of C57BL/6 mice with *L. plantarum* 0111 increased the expression of type I interferon in serum and bronchoalveolar lavage fluid (BALF) and increased the transcriptional levels of IFN-β and ISGs in the intestine and lungs. C57BL/6 mice (*n* = 5/group) were euthanized 7 days after the continuous oral administration of *L. plantarum* 0111 (1 × 10^8^ CFU/200 μl/mouse), and IFN-α and IFN-β expression in serum and bronchoalveolar lavage fluid (BALF) was measured. **(A)** IFN-α (pg/ml) in serum. **(B)** IFN-α (pg/ml) in BALF. **(C)** IFN-β (pg/ml) in serum. **(D)** IFN-β (pg/ml) in BALF. Q-PCR was performed on the intestines and lungs treated differently in different groups of mice. Transcript levels of IFN-α, IFN-β, PKR, MX1, OaS1, and ISG15 were determined. **(E)** Transcription levels of IFN-α, IFN-β, PKR, MX1, OaS1, and ISG15 in the mouse intestine. **(F)** Transcription levels of IFN-α, IFN-β, PKR, MX1, OaS1, and ISG15 in the mouse lungs. PBS: PBS pretreated mice were used. *L. plantarum* 0111: Mice pretreated with *L. plantarum* 0111. MOCK: control group in which no template cDNA was added. The results are presented as the means ± SEMs (*N* = 3), and statistical significance was calculated by *t*-test. ^*^P < 0.05; ^**^P < 0.01; ^***^P < 0.001.

It has been shown that specific microorganisms derived from the gut can resist viral infections by increasing the expression of type I interferons (Yang et al., 2021). Type I interferons play a crucial role in the infection phase and are important in assessing antiviral capacity. The expression levels of IFN-α and IFN-β in serum and BALF were measured in mice after 7 days of continuous oral administration of *L. plantarum* 0111. The results showed that the oral administration of *L. plantarum* 0111 could enhance the expression levels of IFN-β in serum as well as BALF (*p* < 0.05; Figures 6C,D). Notably, the expression levels of IFN-α were not significantly different in either the serum or BALF (Figures 6A,B). Therefore, pretreatment with *L. plantarum* 0111 enhanced the induction of IFN-β but not IFN-α.

The ability of the host to suppress viral infection largely depends on the effectiveness of the initial antiviral innate immune response, which leads to the upregulation of IFNs, followed by ISGs. PKR, MX1, OaS1, and ISG15 are the main antiviral proteins produced by IFN stimulation (Lenschow et al., 2007; Schoggins, 2019). Next, we tested type I interferon and ISG expression after *L. plantarum* 0111 pretreatments. We measured the relative mRNA levels of IFN-α, IFN-β, and the four ISGs in the intestine and lungs of mice by Q-PCR. The results of examining the intestine showed that the transcript levels of MX1 (*p* < 0.05) and ISG15 (*p* < 0.01) were increased by approximately 1.6-fold compared with the control group. At the same time, the transcript level of IFN-β was also elevated (*p* < 0.05; Figure 6F). Notably, the transcript levels of IFN-α, PKR, and OaS1 were not significantly elevated. The results of detecting the lungs showed that the expression of IFN-β was increased by 1.8-fold compared with the control group, while the expression level of ISG15 was also significantly increased in the intestine (*p* < 0.01). Unlike the intestine, the expression level of OaS1 was significantly increased in the lungs compared with the control group (*p* < 0.001). At the same time, the expression of IFN-α, PKR, and MX1 was not elevated in the lungs (Figure 6E).

### *Lactiplantibacillus plantarum* 0111 Regulates T and B Cell Responses

**Figure 7:**
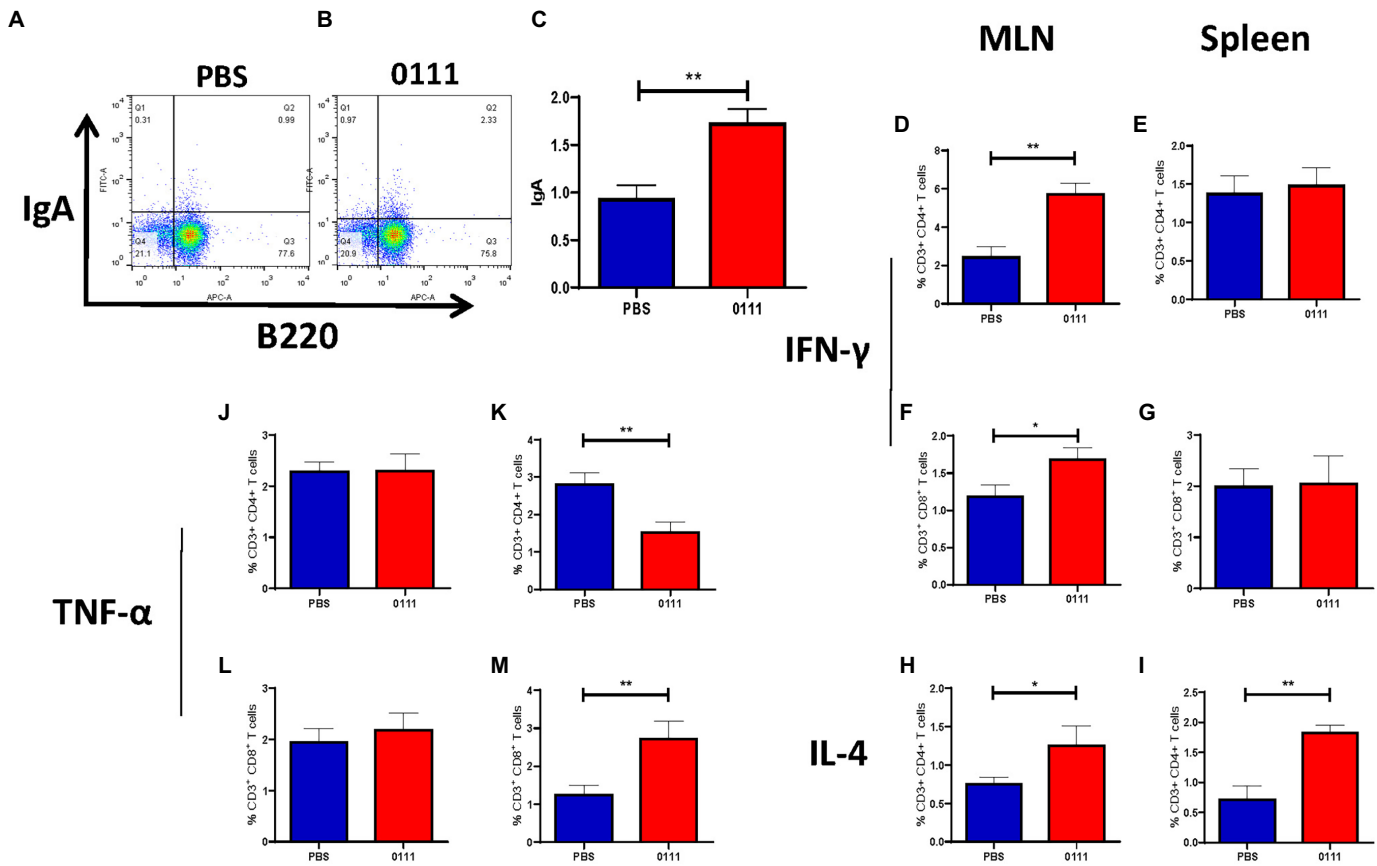
0111 regulates adaptive immunity. C57BL/6 mice (*n* = 5/group) were euthanized by the continuous oral administration of *L. plantarum* 0111 (1 × 10^8^ CFU/200 μl/mouse), single cells were prepared as described, and flow cytometry analysis was performed using the indicated gating method **(A)**. CD4^+^ IFN-γ^+^ T cells **(D)**, CD8^+^ IFN-γ^+^ T cells **(F)**, CD^4^+1L-4^+^ T cells **(H)**, CD4^+^ TNF-α^+^ T cells **(J)**, and CD8^+^ TNF-α^+^ T cells **(L)** in MLNs and CD4^+^ IFN-γ^+^ T cells **(E)**, CD8^+^ IFN-γ^+^ T cells **(G)**, and CD4^+^ 1L-4^+^ T cells **(I)** in the spleen of immunized mice were calculated. The percentages of CD4^+^ TNF-α^+^ T cells **(K)** and CD8^+^ TNF-α^+^ T cells **(M)**. The number of B220^+^ IgA^+^ B cells cells in the Peyer patches of the PBS group **(A)** and *L. plantarum* 0111 group **(B)** as well as statistical analysis **(C)**. The results were calculated for statistical significance using the *t*-test. ^*^P < 0.05; ^**^P < 0.01.

The activation and polarization of T cells in MLNs and spleen were detected by flow cytometry (Supplementary Figure S2). The 0111 group significantly increased the percentages of CD3^+^ CD4^+^ IFN-γ^+^ T cells (Figure 7D; *p* < 0.01) and CD3^+^ CD8^+^ IFN-γ^+^ T cells (Figure 7F; *p* < 0.05) in MLNs compared with the PBS group, although a similar trend was not observed in the spleen (Figures 7E,G). On the other hand, the 0111 group had an increased percentage of CD3^+^ CD4^+^ IL-4^+^ T cells in both the MLNs (*p* < 0.05) and spleen (*p* < 0.01; Figures 7H,I). In addition, the 0111 group significantly increased the percentages of CD3^+^CD4^+^TNF-α^+^ T cells (Figure 7K; *p* < 0.01) and CD3^+^CD8^+^ TNF-α^+^ T cells (Figure 7M; *p* < 0.01) in the spleen compared with the PBS group, while no difference was observed in the MLNs (Figures 7J,L). The numbers of B220^+^ IgA^+^ B cells in Peyer’s patches were examined. The number of B220^+^ IgA^+^ B cells (*p* < 0.01) was significantly increased (Figures 7A–C), demonstrating that 0111 had a regulatory effect on cytokines within T cells and increased the expression of IgA within B cells in the MLN and spleen.

## Correlation Analysis

**Figure 8:**
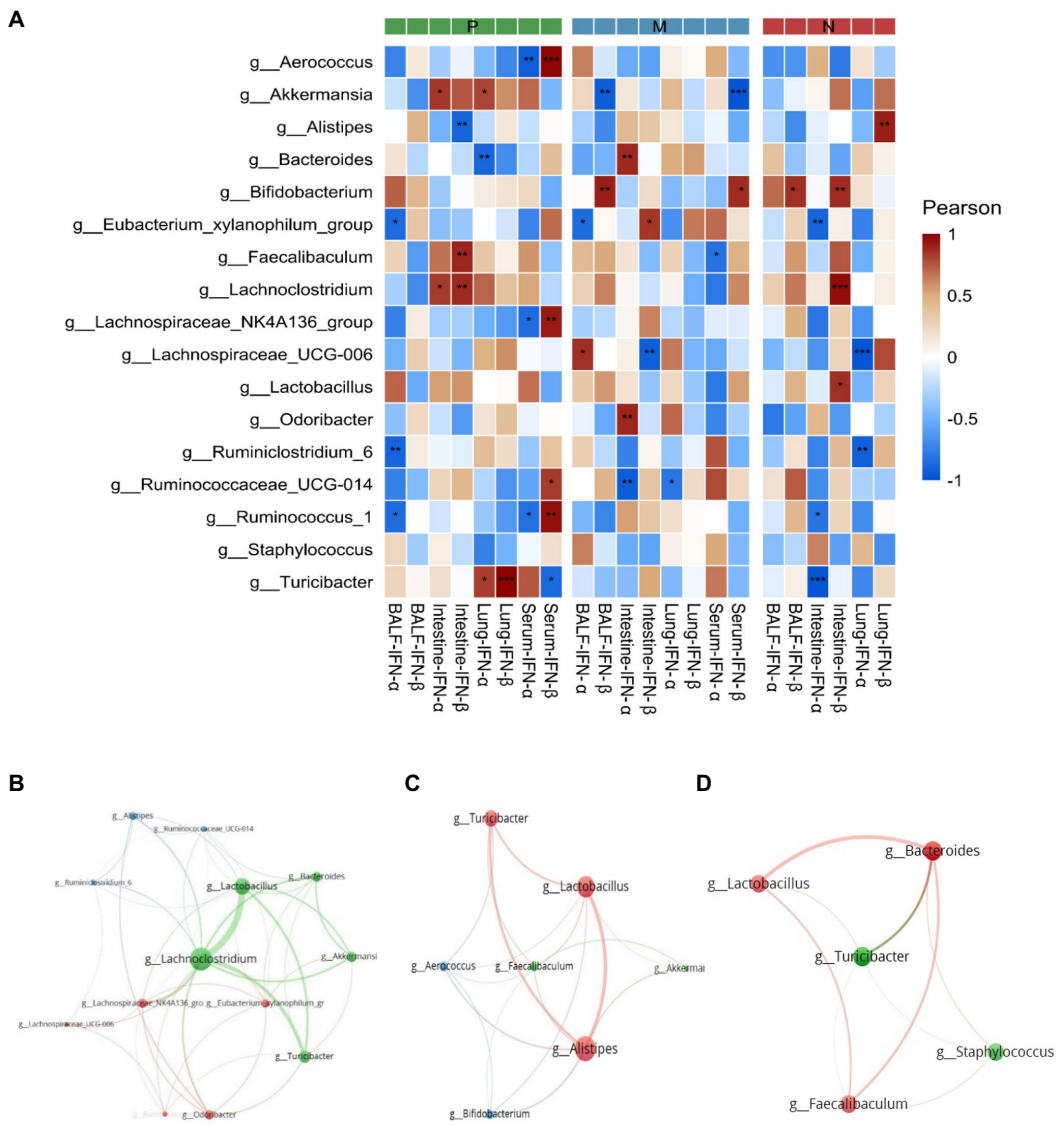
The Pearson correlation between dominant bacterial genera and the transcription and expression of type I interferon **(A)** and the correlation between the dominant bacterial genera in groups P **(B)**, M **(C)**, and N **(D)** are shown in the correlation diagram. The *P* values were adjusted according to the Bonferroni and Hochberg procedures to determine the false discovery rate. Asterisks indicate a significant correlation between bacterial groups and type I interferon (^*^P < 0.05, ^**^P < 0.01, ^***^P < 0.001). P, challenged H9N2 cells after pretreatment with PBS; M, challenged H9N2 cells after pretreatment with *L. plantarum* 0111; N, pretreatment with PBS.

Pearson correlation results are shown in Figure 8A. At the expression level of type I interferon, *Ruminococcus-1*, *Lachnospiraceae-NK4A136*-group, and *Aerococcus* in group P were significantly positively correlated with serum IFN-β expression negatively correlated with serum IFN-α expression, while *Turicibacter* also showed a negative correlation with serum IFN-β. *Ruminiclostridium-6*, *Ruminococcus-1*, and *Eubacterium-xylanophilum*-group were significantly negatively correlated with IFN-α expression in BALF. Akkermansia was negatively correlated with IFN-β in serum and BALF in group M. In contrast, *Bifidobacterium* was positively correlated with IFN-β in serum and BALF. Faecalibaculum and Lachnoclostridium in group P were positively correlated with intestinal IFN-β transcript levels at the transcriptional level, while *Alistipes* was negatively correlated with them. Notably, there was a significant positive correlation between *Turicibacter* and IFN-β transcript levels in the lungs. In group M, *Bacteroides* and *Odoribacter* were positively correlated with intestinal IFN-α transcript levels, while *Ruminococcaceae UCG-014* was significantly negatively correlated with intestinal and lung IFN-α transcript levels. In addition, correlation networks were plotted for *Actinobacteria*, *Verrucomicrobia*, *Firmicytes*, and *Bacteroidetes* to show the relationships among some important members belonging to the four dominant phyla (Figures 8B–D).

## Discussion

Here, we demonstrate that oral administration of *L. plantarum* 0111 can prevent and protect against H9N2 influenza virus infection in mice. We also reveal that oral administration of *L. plantarum* 0111 can maintain the balance of intestinal microbes and modulate the immune response to mitigate the aggression caused by the influenza virus.

Viral infection heavily relies on host transfer RNA (tRNA) for viral RNA decoding, which is a major determinant of the success of viral infection (Ou et al., 2020). Factors controlling tRNA use in the intestine remain unexplored, but microbiome and virome can influence this, and thus by inference, *L. plantarum* could as well (Zhang et al., 2021). There is increasing evidence that probiotics provide immune protection by regulating multiple aspects of the innate and adaptive immune systems (Giorgetti et al., 2015). The potential antiviral mechanisms of probiotics in the enteropulmonary axis and mucosal immune system may play a role in the prevention and supportive treatment of SARS-CoV-2 infection (Sundararaman et al., 2020). Therefore, probiotics may be involved in activating immune responses and actively participate in the protection of respiratory tract infections. In the present study, mice orally administered *L. plantarum* showed excellent protection against lethal influenza virus infection, as indicated by reduced weight loss and viral load in the lungs and improved survival. Thus, *L. plantarum* 0111, a new Lactobacillus strain, may have anti-influenza virus activity.

The main role of commensal bacteria is to maintain normal homeostasis by regulating the host’s immunity, development, and physiology (Yitbarek et al., 2018). Microorganism-associated molecular patterns (MAMPs) of bacteria, including lipopolysaccharide, flagellin, peptidoglycan, formyl peptides and unique nucleic acid structures, as well as gut microbe-derived SCFAs, tryptophan metabolites, and polyamines, can modulate the gut immune system (Rooks and Garrett, 2016). H9N2-type AIV infection can cause dysbacteriosis in the intestine (Li et al., 2018). Probiotic supplementation enables probiotics to dominate the gut, maintaining gut health (Sanders et al., 2019). A mathematical analysis predicts that the diversity of microbial communities in the gut may influence the stability of the intestinal system (Coyte et al., 2015). The 16S sequencing results showed that H9N2 infection altered the gut microbial structure in this study. *Lachnoclostridium*, *Ruminococcaceae-UCG-014*, *Ruminococcus-1*, *Lachnospiraceae-UCG-006*, *Eubacterium-xylanophilum*-group, *Ruminiclostridium-6*, *Odoribacter*, and *Lachnospiraceae-NK4A136*-group were only present in the H9N2-infected group and not in the supplemented 0111 group. *Faecalibaculum* is beneficial for intestinal health and has been shown to have antitumor properties (Li et al., 2020; Zagato et al., 2020). Supplementation with *L. plantarum* 0111 restored the dramatic decreases in the abundances of *Faecalibaculum* and *Lactobacillus* caused by H9N2 infection, thus maintaining the balance of intestinal microorganisms. It has been shown that *Alistipes* is a pathogenic factor in colorectal cancer, and deregulation of *Alistipes* abundance affects the intestinal microenvironment (Parker et al., 2020). In this study, H9N2 infection resulted in an elevation of *Alistipes* abundance and was inversely correlated with the transcript level of intestinal IFN-β. Notably, supplementation with *L. plantarum* 0111 significantly reduced *Alistipes* abundance. A commensal bacterium of the mucus layer of *Akkermansia* can regulate metabolism, immune responses, and gut health protection (Zhai et al., 2019). *Akkermansia* is the most important propionic acid producer and an important modifier of immunity, both in humans and mice (Al-Lahham et al., 2010; Su et al., 2021). *Akkermansia*-related protein enrichment regulates the intestinal barrier by activating TLR2 and TLR4 to induce the production of specific cytokines (Ottman et al., 2017). Thus, *L. plantarum* 0111 may act synergistically with other probiotics to activate the immune system.

The pathogenesis of viral infection is associated with an excessive inflammatory response (de Jong et al., 2006; Wang et al., 2010). It has been reported that during influenza virus infection, a large number of inflammatory cells infiltrate the lungs and produce excessive inflammatory cytokines, forming a cytokine storm resulting in severe lung injury (Kobasa et al., 2007; Cillóniz et al., 2009). A significant reduction in IL-6 production in the lungs was found after administering *Bifidobacterium bifidum* to mice infected with lethal influenza A (H1N1; Iwabuchi et al., 2011). Type I interferons play an important role in antagonizing viral replication, monitoring the immune system, and inhibiting cytopathic effects as a powerful tool for the host to protect against infection by foreign pathogens (Fuertes et al., 2013; Rönnblom, 2016). After invading the body, the influenza virus activates the host’s innate immune response and induces the production of type I interferon, which then binds to its receptors IFNAR1 and IFNAR2 and activates downstream signaling pathways, thereby activating the transcription and expression of downstream ISGs (Pestka, 2007). In addition to canonical ISG transcriptional regulation mechanisms, non-canonical ISG transcriptional regulation plays an important role (Wang et al., 2017). For example, bacteria can affect the transcription of ISGs by altering epigenetic regulators of transcription (Alphonse et al., 2021). The expression products of ISGs can directly promote the antiviral effect of the body and further induce type I interferon, thereby indirectly enhancing the antiviral effect of the body (Schoggins, 2014). Many ISGs, OAS1, Mx1, PKR, and ISG15, are common ISGs (Schoggins, 2019). Previous studies have shown that probiotics can reduce the viral load and thus exert a protective effect (Goto et al., 2013; Park et al., 2013). *Lactococcus lactis* JCM5805 and *Lactobacillus gasseri* SBT2055 increased the expression of antiviral factors in the lung, which is similar to our findings (Nakayama et al., 2014; Jounai et al., 2015). In the present study, the transcription levels of IFN-β, OAS1, Mx1, and ISG15 in the lungs and intestines of mice pretreated with *L. plantarum* 0111 were correspondingly increased, and the lung viral load was significantly reduced after viral infection. Therefore, the probiotic regulatory pathway may depend on the natural immune system and is closely linked to type I interferons.

In diseased individuals, the ability of commensal gut bacteria to modulate the host immune system has been demonstrated (Perez-Lopez et al., 2016). Commensal bacteria also modulate regulatory T cells by recognizing the bacteria themselves or their metabolites/products by immune cells and greatly affect mucosal immunity (Hall et al., 2008; Goto et al., 2016). Probiotic supplementation of healthy neonates significantly enhanced IFN-γ expression, indicating that probiotics play an important role in improving Th1 immune responses. In addition, *bifidobacteria* were shown to have powerful potential to modulate humoral and cellular immune responses and induce a balanced Th1/Th2 immune response against influenza infection (Mahooti et al., 2019). Our study determined the differentiation direction of Th cells into Th1 and Th2 subtypes by the percentage of IFN-γ and IL-4 produced by CD4^+^ T cells. The results showed that the expression of IFN-γ and IL-4 in TH cells was increased to different extents after treatment with *L. plantarum* 0111, indicating their potential regulatory effects on humoral and cellular immunity. IFN-γ can induce virus-infected cells to express viral antigens, increase the ability of the immune system to recognize and kill infected cells and resist viral replication (Maggi et al., 1992). NK cells are early antiviral effector cells that can directly kill infected cells. IL-12 can promote the proliferation of NK and Th1 cells, and the proliferation of Th1 cells may also affect the proliferation of NK cells to resist viral infection. In addition, IFN-γ produced by Th1 cells could further activate NK cells, suggesting the possibility of NK cells exerting antiviral effects (Komastu et al., 1998). In the meantime, it has been shown that probiotics elevate IgA expression in B cells within gut-associated lymph nodes and intestinal secretory IgA (Kikuchi et al., 2014). The results of this experiment are consistent with these results, suggesting that IgA is essential for intestinal protection at the initial stage of influenza virus infection.

In summary, *L. plantarum* 0111 protects mice from damage caused by H9N2, and this protection may be a function of the innate and acquired immune response mediated by the gut microbiota. Therefore, *L. plantarum* 0111 could be a candidate strain to prevent the influenza virus.

## Data Availability Statement

The datasets presented in this study can be found in online repositories. The name of the repository and accession number can be found at: NCBI Sequence Read Archive; SRP301018.

## Ethics Statement

The animal study was reviewed and approved by Animal Management and Ethics Committee of Jilin Agricultural University (JLAU20200704001).

## Author Contributions

WTY, GLY, and C-FW contributed to the conception of the study. J-HX, C-WS, M-JS, WG, R-RZ, and H-LC contributed significantly to analysis and manuscript preparation. YL, DW, JYL, T-MN, Q-TH, and J-HQ performed the data analyses and wrote the manuscript. HBH, Y-LJ, J-ZW, XC, NW, and YZ helped perform the analysis with constructive discussions. All authors contributed to the article and approved the submitted version.

## Funding

This work was supported by the National Natural Science Foundation of China (31941018, 31972696, 32072888, and U21A20261), the China Agriculture Research System of MOF and MARA (CARS-35), the Science and Technology Development Program of Jilin Province (20190301042NY YDZJ202102CXJD029, and 20210202102NC), and the Science and Technology Research Program of Education Department of Jilin Province (JJKH20220365KJ).

## Conflict of Interest

WG was employed by Shandong BaoLai-LeeLai Bioengineering Co. Ltd.

The remaining authors declare that the research was conducted in the absence of any commercial or financial relationships that could be construed as a potential conflict of interest.

## Publisher’s Note

All claims expressed in this article are solely those of the authors and do not necessarily represent those of their affiliated organizations, or those of the publisher, the editors and the reviewers. Any product that may be evaluated in this article, or claim that may be made by its manufacturer, is not guaranteed or endorsed by the publisher.

## Supplementary Material

The Supplementary Material for this article can be found online at: https://www.frontiersin.org/articles/10.3389/fmicb.2022.820484/full#supplementary-material
