# Supplementary material for: Lactiplantibacillus plantarum 0111 Protects Against Influenza Virus by Modulating Intestinal Microbial-Mediated Immune Responses
Source: Front Microbiol. 2022 Jun 30;13:820484. doi: 10.3389/fmicb.2022.820484 (PMC9282045; doi:10.3389/fmicb.2022.820484)
Supplement: Supplementary file 1 [file Data_Sheet_1.docx]

***Lactiplantibacillus plantarum* 0111 protects against influenza virus by modulating intestinal microbial-mediated immune responses**

Jun-Hong Xing ^1†^, Chun-Wei Shi ^1†^, Ming-Jie Sun ^1^, Wei Gu ^2^, Rong-Rong Zhang ^1^, Hong-Liang Chen ^1^, Ying Li ^1^, Dan Wang ^1^, Jun-Yi Li ^1^, Tian-Ming Niu ^1^, Qun-Tao Huang ^1^, Jia-Hao Qian ^1^, Hai-Bin Huang ^1^, Yan-Long Jiang ^1^, Jian-Zhong Wang ^1^, Xin Cao ^1^, Nan Wang ^1^, Yan Zeng ^1^, Wen-Tao Yang ^1,*^ , Gui-Lian Yang ^1,*^, Chun-Feng Wang ^1,*^

^1^ College of Veterinary Medicine, College of Animal Science and Technology, Jilin Provincial Engineering Research Center of Animal Probiotics, Key Laboratory of animal production and product quality safety of Ministry of Education, Jilin Agricultural University, Changchun, China.

^2^ Shandong BaoLai-LeeLai Bioengineering Co. Ltd, Tai'an, 271000, Shandong, China.

* Corresponding authors:

Chun-Feng Wang, College of Animal Science and Technology, College of Veterinary Medicine, Jilin Provincial Engineering Research Center of Animal Probiotics, Jilin Agricultural University, 2888 Xincheng Street, Changchun 130118, China. E-mail: wangchunfeng@jlau.edu.cn, Tel./fax: +86 43184533426.

Wen-Tao Yang, College of Animal Science and Technology, College of Veterinary Medicine, Jilin Provincial Engineering Research Center of Animal Probiotics, Jilin Agricultural University, 2888 Xincheng Street, Changchun 130118, China. E-mail: yangwentao@jlau.edu.cn.

Gui-Lian Yang, College of Animal Science and Technology, College of Veterinary Medicine, Jilin Provincial Engineering Research Center of Animal Probiotics, Jilin Agricultural University, 2888 Xincheng Street, Changchun 130118, China. E-mail: yangguilian@jlau.edu.cn, Tel./fax: +86 43184533425.

† These authors have contributed equally to this work

**Running Title:** The protective effect of *Lactiplantibacillus plantarum* 0111 on mice.

[**Table.S1**](http://www.jmb.or.kr/journal/popup_file.html?uid=5394&file=JMB2030-04-515_Fig_01.jpg&md=tbl&idx=1).Q-PCR primers（Mouse） used in the present study

| **Gene name （**Mouse**） Primer sequences (5’-3’)** |
| --- |
| IFN-β Forward TGTCCTCAACTGCTCTCCACTTGAA  Reverse TCCAGGCGTAGCTGTTGTACTTCAT  IFN-α Forward GAATTTCCCCTGACCCAGGAAGATG  Reverse AGGGGCTGTGTTTCTTCTCTCTCAG  PKR Forward CGTGCTTCACGGAGTCAGAGAAAAT  Reverse TTCAGATGTCTCAGGTCGGTCCTTG  Mx1 Forward ACGGTGCAGACATACCAGAAGATGA  Reverse TGTCTCCCTCTGATACGGTTTCCTG  Oas1 Forward TTTAAGTACAGGGACGGTTCCCCAG  Reverse GGGCTATCCAGATGAAGTCTTCCCA  ISG15 Forward ACGGTGTCAGAACTGAAGAAGCAGA  Reverse CCAGACCCAGACTGGAAAGGGTAAG  β-actin Forward GATCAAGATCATTGCTCCTCCTG  Reverse AGGGT GTAAAACGCAGCTCA |

[**Supplementary**](javascript:;) [**method**](javascript:;)

Briefly, predenaturation was performed at 94 °C for 3 min, followed by 25 cycles of denaturation at 94 °C for 30 s, annealing at 50 °C for 30 s and extension at 72 °C for 60s. The final extension step was performed at 72 °C for 7 min. PCR was performed in triplicate in 25μL volumes containing 2.5μl of 10× Pyrobest Buffer (TaKaRa, Shiga, Japan), 2 μl of 2.5mM d NTPs, 1μl of each primer (10μM), 0.4 U of Pyrobest DNA polymerase (TaKaRa, Shiga, Japan), and 15ng of template DNA. PCR products were run in an electrophoresis chamber on a 2% agarose gel, purified using an AxyPrep DNA Gel Extraction Kit (Axygen Biosciences, Union City, CA, USA) following the manufacturer’s instructions and quantified using QuantiFluor™-ST (Promega, Madison, Wisconsin, USA). Purified amplicons were used for library preparation and pyrosequencing. Sequencing libraries were generated using an NEB-Next® Ultra™ DNA Library Prep Kit (New England Bio-labs, Ipswich, MA, USA) following the manufacturer’s recommendations. Library quality was assessed, and libraries were sequenced on an Illumina MiSeq PE300 platform (Illumina, Inc., CA, USA).

**d**

**e**

**f**

**a**

**b**

**c**

**Fig. S1** Pretreatment of mice with *Lactiplantibacillus plantarum* 0111 provided protection against H9N2 influenza virus infection. C57BL/6 mice (n = 5 per group) were pretreated orally with different doses of *Lactiplantibacillus plantarum* 0111 7 days prior to H9N2 influenza virus infection (a) Body weight changes in mice pretreated with *Lactiplantibacillus plantarum* 0111 (1 × 10^7^ CFU/50 ul/mouse) after infection with H9N2 virus. (b) Body weight changes in mice pretreated with *Lactiplantibacillus plantarum* 0111 (1 × 10^8^ CFU/50 ul/mouse) after infection with H9N2 virus. (c) Body weight changes in mice pretreated with *Lactiplantibacillus plantarum* 0111 (1 × 10^9^ CFU/50 ul/mouse) after infection with H9N2 virus. (d) Survival of mice pretreated with *Lactiplantibacillus plantarum* 0111 (1 × 10^7^ CFU/50 ul/mouse) after infection with H9N2 virus. (d) Survival of mice pretreated with *Lactiplantibacillus plantarum* 0111 (1 × 10^8^ CFU/50 ul/mouse) after infection with H9N2 virus. (f) Survival of mice pretreated with *Lactiplantibacillus plantarum* 0111 (1 × 10^9^ CFU/50 ul/mouse) after infection with H9N2 virus.


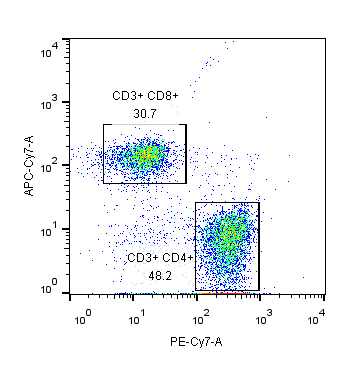

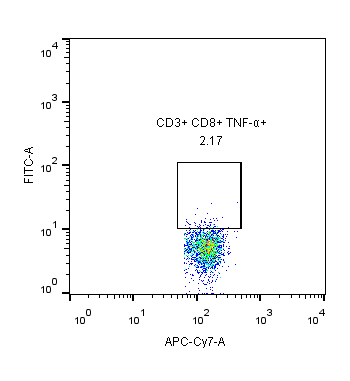

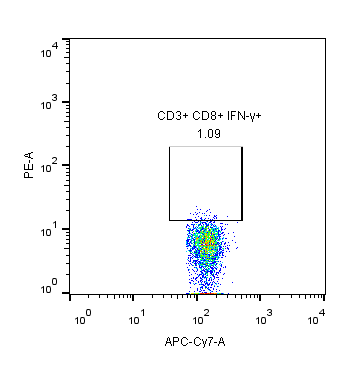

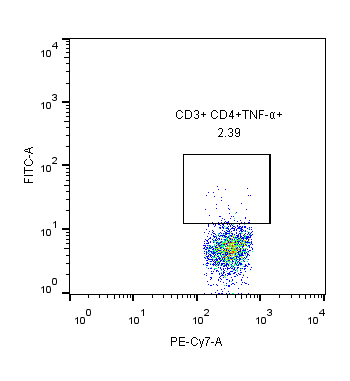

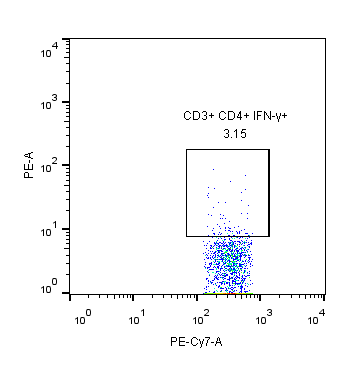

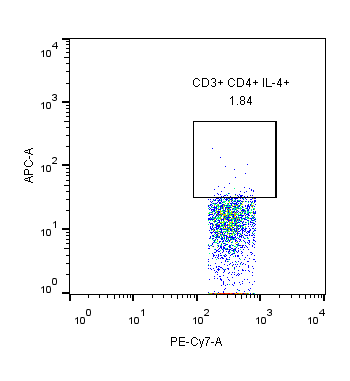

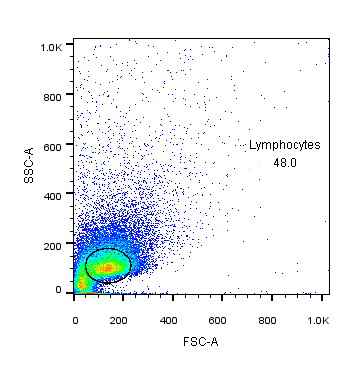

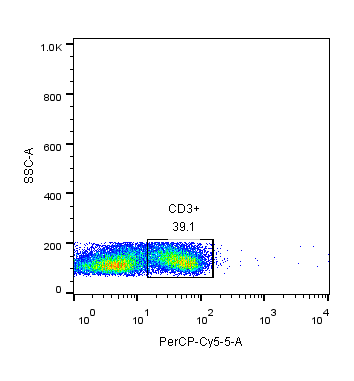


**Fig. S2** *Lactiplantibacillus plantarum* 0111 regulates T cell response. C57BL/6 mice (n = 5/group) were euthanized by continuous oral administration of *Lactiplantibacillus plantarum* 0111 (1 × 10^8^ CFU/200 μL/mouse), single cells were prepared as described, and flow cytometry analysis was performed using the indicated gating method.


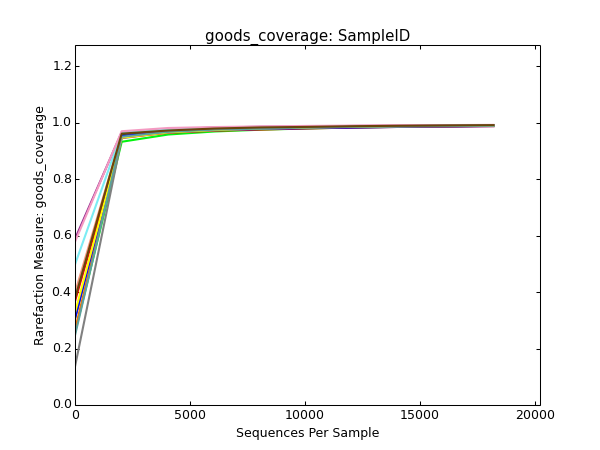

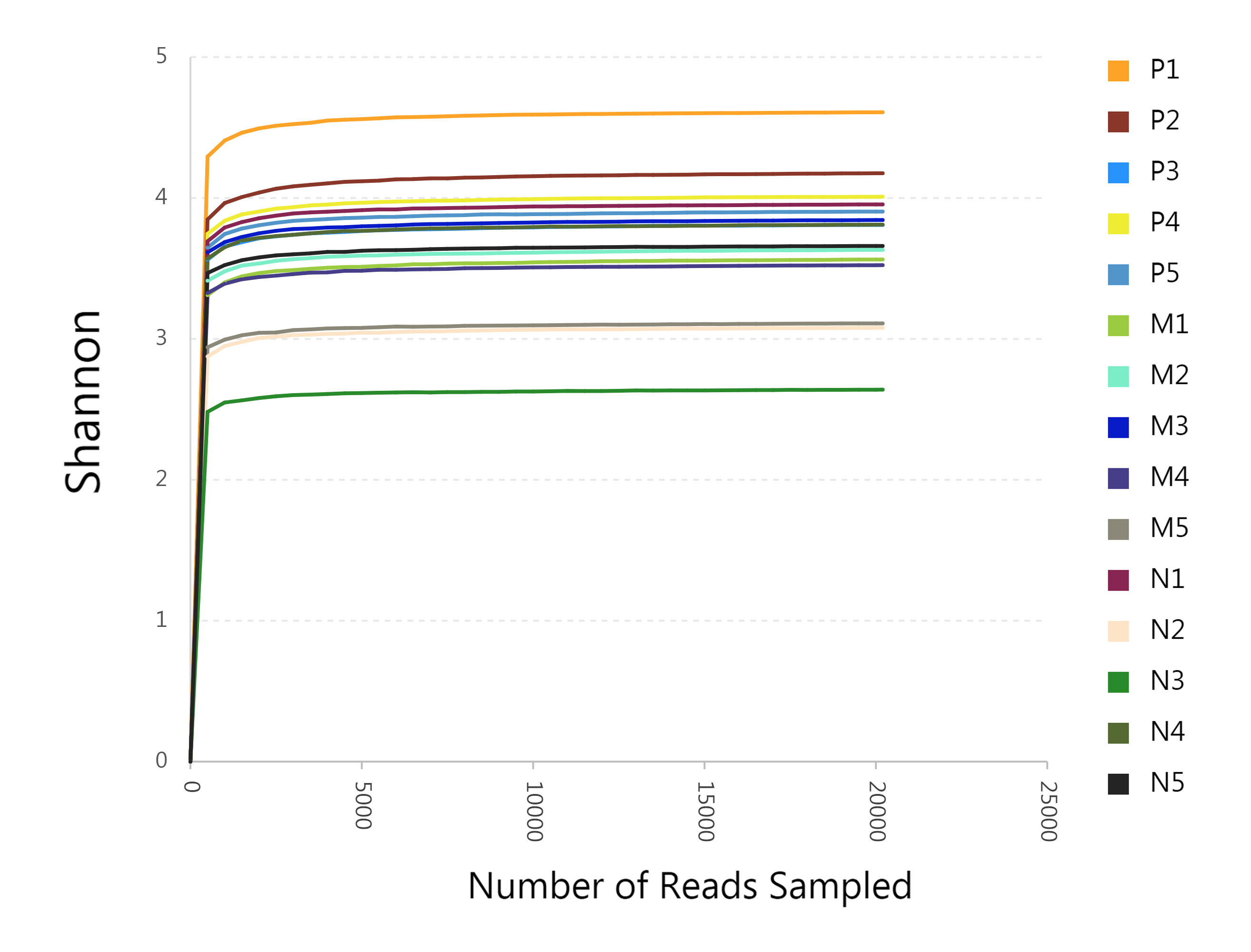

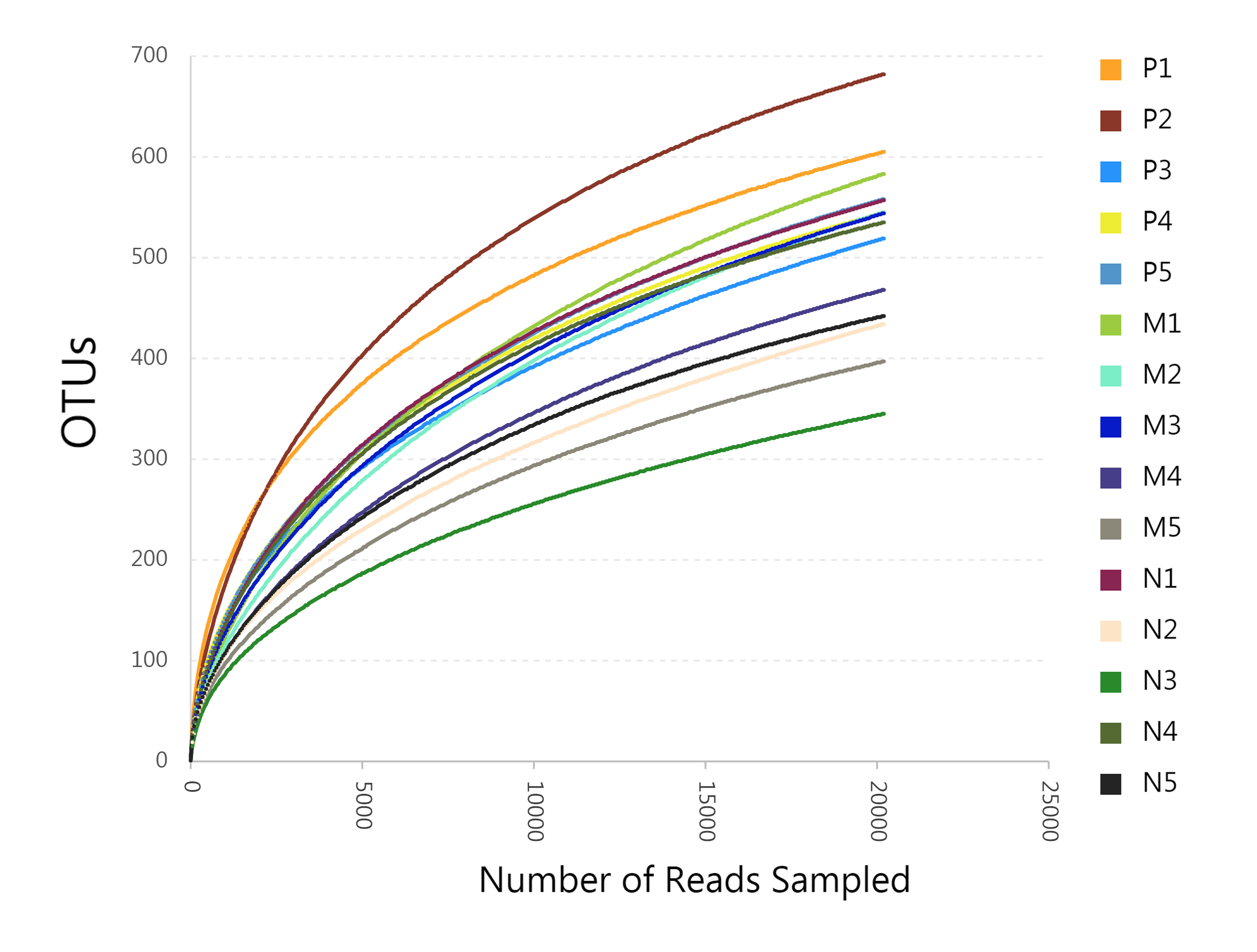

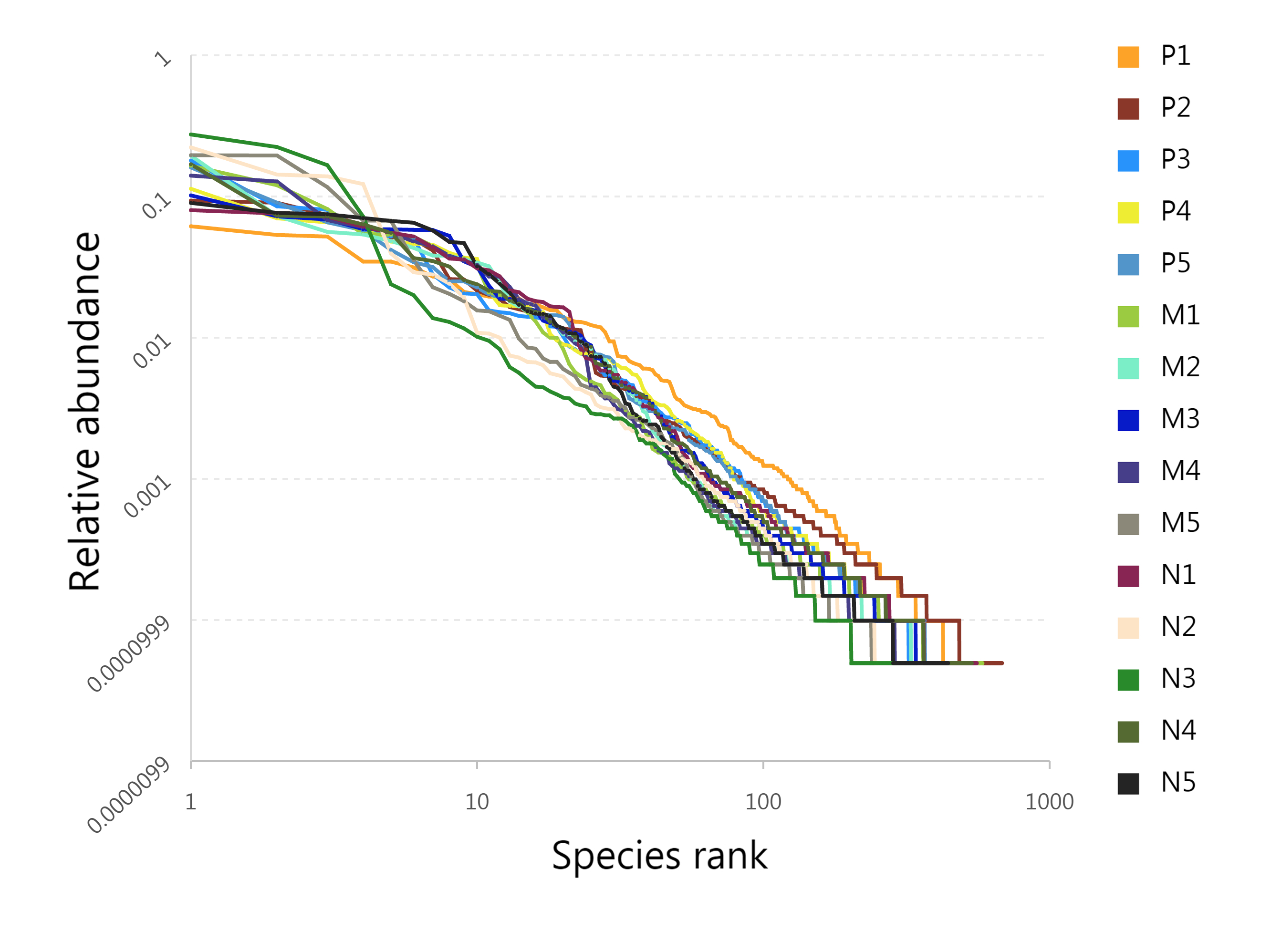


**a**

**d**

**c**

**b**

**Fig. S3** After sequencing analysis of the 16S rRNA gene, the OTUs curves obtained from the three groups. (a) Good's coverage coverage curves; (b) Rarefaction curves; (c) Shannon - Wiener curves; (d) Species accumulation curves. Abbreviation: P, challenge H9N2 after pretreatment with PBS; M, challenge H9N2 after pretreatment with *Lactiplantibacillus plantarum* 0111. N, pretreatment with PBS.


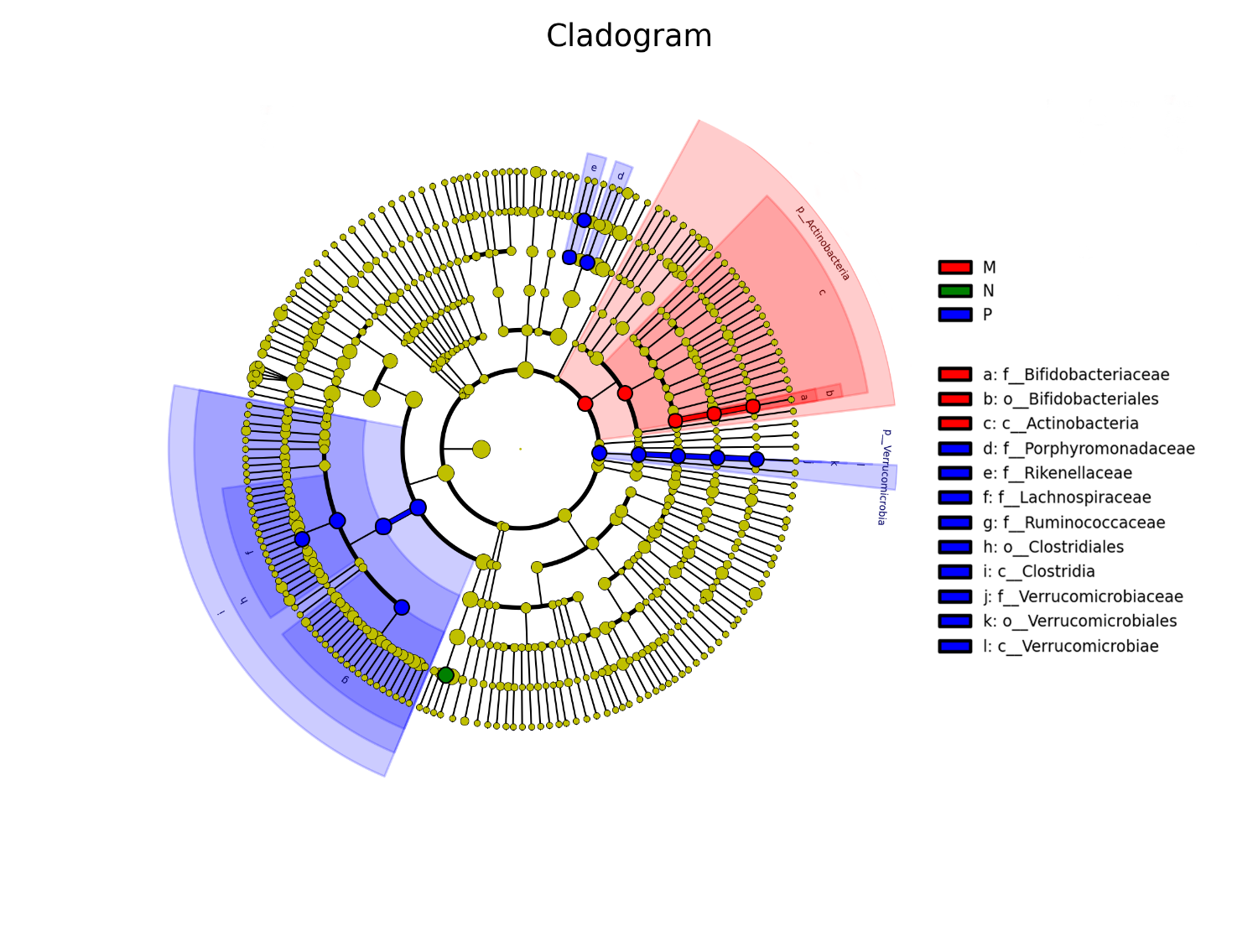


**Fig. S4** Cladogram of the LEfSe analysis of the gut microbiota in different groups. The microbial compositions were compared at different evolutionary levels. Abbreviation: P, challenge H9N2 after pretreatment with PBS; M, challenge H9N2 after pretreatment with *Lactiplantibacillus plantarum* 0111. N, pretreatment with PBS.
